# A European Blueprint for Community Nursing: Building Care Protocols on International Organization for Standardization (ISO) 7101:2023

**DOI:** 10.7759/cureus.112021

**Published:** 2026-07-03

**Authors:** Giuseppe Fumai, Vincenzo Abbasciano, Giovanna Muto, Nunzia Cappucci, Carla Recchia

**Affiliations:** 1 Orthopedic and Neurological Rehabilitation, Rehcura Rehabilitation Facility, Adelfia, ITA; 2 Biomedical Sciences, University of Foggia, Bari, ITA; 3 Internal Medicine/Wound Care, Local Health Authority Andria, Andria, ITA; 4 Cardiothoracic Surgery, University of Padua, Padua, ITA; 5 Complex Administrative-Legal Framework, Local Health Authority Foggia, Foggia, ITA; 6 Biomedical Sciences, University of Foggia, Foggia, ITA

**Keywords:** care protocol standardization, community nursing, district nursing, family and community nurse, healthcare quality management, iso 7101, people-centered care, primary health care

## Abstract

Care across Europe is moving out of the hospital and into homes and communities. The family and community nurse now stands at the center of this shift, holding continuous responsibility for people where they live. Yet the procedures that guide this work are often written locally and vary widely, with no shared quality architecture behind them. This editorial proposes International Organization for Standardization (ISO) 7101:2023 as a possible common European reference model for designing community nursing care protocols, anchoring each procedure to quality principles such as people-centered care, co-production, risk control, and continual improvement rather than to ad hoc local practice. We develop the case across Europe, including the United Kingdom, where community and district nursing is well established within the National Health Service. We then propose a five-phase heuristic model that turns each procedure into a small, people-centered quality system anchored to the standard. Adopting this approach would curb unwarranted variation, strengthen patient safety, and make community care auditable and open to improvement. The editorial offers an evidence-informed normative proposal rather than an empirical study; the model is preliminary and will require future validation.

## Editorial

Healthcare in Europe is moving out of the hospital. Aging populations, the rise of chronic disease, and sustained pressure on acute beds are pushing care toward homes, local clinics, and the community. At the center of this shift stands the community nurse. This is the professional who takes continuous responsibility for people where they live, carrying the everyday work of prevention, monitoring, and continuity. The role is now being formalized. A standardized professional profile for the family and community nurse has been defined across Europe through agreed core competencies, and several national systems have begun aligning their own profiles with it [1]. The competencies of the nurse are converging. The procedures the nurse follows are not.

Here lies the structural problem. A nursing procedure is the operational unit of safe care. It sets out what is done, by whom, with what safeguards, and how it is recorded. In community settings, these procedures are too often written locally, borrowed from hospital documents, and rarely anchored to a shared quality framework. Quality governance itself remains fragmented and regionally uneven, and the evidence evaluating it is thin [2]. The result is unwarranted variation. The same clinical need is met with different procedures, different safety controls, and different records from one district to the next. Care delivered far from institutional oversight is precisely the care most exposed to this drift.

The community setting sharpens the stakes. Hospital care is wrapped in layers of supervision, with colleagues, protocols, and rapid escalation close at hand. The community nurse often works alone, in the patient’s home, far from immediate support. Oversight is lighter, and the environment is uncontrolled. Here, the written procedure carries more of the safety load, not less. A weak or missing protocol is felt directly at the bedside. A strong one becomes the nurse’s main structural defense against error.

An asymmetry drives the problem. The role is being standardized at the level of competencies, through shared European profiles, while the procedures that put those competencies into practice are left to local improvisation [1]. The system has agreed on what the nurse should be able to do. It has not been agreed how the work itself should be built, governed, and improved. This imbalance discourages systematic investment in the quality of procedures. It leaves frontline nurses to reinvent safe practice, document by document, district by district.

A new instrument now makes a different approach possible. International Organization for Standardization (ISO) 7101:2023 is the first global consensus standard for healthcare quality management [2,3]. It applies to any organization that delivers care, whatever its size or setting. Its logic marks a deliberate move away from compliance pursued for its own sake. The standard organizes quality around people-centered care, co-production with service users, equity, risk-based thinking, and continual improvement. These are not abstract values. They are requirements that an organization must put into operation and be able to demonstrate. For community nursing, the relevance is direct. The principles of the standard map closely onto what a sound community procedure should contain.

Early evidence suggests that this translation works at the point of care. In a recent implementation study, a single health-literacy requirement of ISO 7101 was turned into a standardized checklist and delivered by trained nurses during consultations [4]. Patients’ general health literacy scores then rose from 66.35 to 76.29, a statistically significant gain with a large effect size [4]. The mechanism is what matters. A principle in the standard became a concrete nursing procedure, and that procedure produced a measurable improvement in a patient-centered outcome. A community nursing protocol would follow the same path, scaled from a single clause to a complete care pathway.

The United Kingdom offers a mature reference from outside the European Union. Its National Health Service has long treated community and district nursing as a structural role, and national workforce standards now guide how these services are staffed and planned [5]. The role and the setting are well established. Yet the procedures these nurses follow are still not anchored to a shared international quality standard. The gap is therefore not confined to systems that are only now building the role. It persists in the most developed ones. Comparable expansion of community and primary care is underway across the rest of Europe, and each system faces the same unanswered question. On what quality architecture should community nursing procedures be built? ISO 7101 is a credible answer. Adopting it now would let services start from a shared standard rather than retrofitting one later.

To make the proposal usable, we set out a heuristic for building community nursing protocols on the standard. The model treats each procedure as a small quality system rather than a static document. Its five phases are not a new framework. They follow the requirement areas of ISO 7101 itself, from the context of the organization through people-centered and co-produced care, the planning of risk and safety, supported delivery, and performance evaluation [3]. The model is cyclical because the standard is. ISO 7101 requires continual improvement, and that requirement turns the final phase back into the first [3]. Each phase converts one domain of the standard into a concrete design action for the nurse. We call it the ISO 7101 community-protocol cycle (Table 1).

**Table 1:** The ISO 7101 community-protocol cycle - a five-phase heuristic for designing community nursing care protocols. Authors’ own elaboration mapping the requirement areas of ISO 7101:2023 onto the design of a community nursing protocol [3]. The sequence follows the structure of the standard, from the context of the organization to continual improvement. The standard’s own requirement for continual improvement returns the final phase to the first. ISO, International Organization for Standardization

Design phase (ISO 7101 anchor)	Application to the community nursing care protocol
1. Context - organizational context and health equity	Define the protocol’s scope from a structured assessment of community and population needs. Identify access barriers and equity gaps in the catchment area.
2. Co-production - people-centered care and co-production	Design the procedure together with patients, families, and community actors. Build in shared decision points and patient-held information.
3. Risk control - risk-based thinking and patient safety	Map the clinical risks specific to home and community delivery. Embed safety checks, escalation criteria, and continuity safeguards.
4. Standardized delivery - operation, support, and competence	Specify the care actions, role boundaries, required competencies, and the minimum dataset recorded at each contact.
5. Improvement - performance evaluation and continual improvement	Define outcome and experience indicators. Audit adherence and revise the protocol on a fixed review cycle.

Read in sequence, the five phases turn an abstract standard into a repeatable design routine. The nurse need not master the full text of ISO 7101 to apply it. The cycle carries the logic of the standard into everyday practice, and it makes every protocol auditable against the standard’s own criteria wherever it is used. Variation that is clinically justified remains. Variation that is merely accidental is designed out.

Putting this into practice will face real barriers. Few nurse educators and clinical leads currently combine community nursing expertise with formal quality-management training. Genuine co-production demands time and structured methods that services do not always have. Audit and continual improvement call for indicators, data, and protected hours. Digital maturity and the pace of reform also differ widely across European systems. None of these obstacles is trivial. Phased adoption, shared protocol templates, and investment in the quality skills of community nurses are pragmatic ways forward. A dedicated feasibility and cost analysis remains a necessary next step.

This editorial advances an evidence-informed normative proposal, not a validated intervention. The model in Table 1 has not been tested for feasibility, uptake, or effect on outcomes in any community setting. The supporting evidence remains limited. One implementation study shows that a single ISO 7101 requirement can become an effective nursing procedure [4], yet no study has evaluated the standard as a framework for entire community nursing protocols. Generating that evidence is the priority. Future research should pilot ISO 7101-based protocols in real community services and measure their effect on variation, patient safety, and patient experience with validated outcome measures.

The move of care into the community is not a temporary adjustment. It is the direction of European health systems. Quality cannot be left to chance while that move accelerates. The community nurse stands where clinical judgment, patient autonomy, and system safety meet, and the procedures that guide this work should carry a quality standard worthy of it. ISO 7101 already provides the international reference. What is missing is its translation into the design of community nursing care. The present wave of reform is the moment to make that translation, before accidental variation hardens into the norm. Doing it well will require universities, professional bodies, and health authorities to move together. The cost of inaction is counted in avoidable harm, and in care whose quality depends on the postcode where it is delivered.

The key message is that ISO 7101:2023 should become the shared European standard for designing community nursing care protocols, because quality in community care is now a structural requirement rather than an optional extra.
